# Physical Activity or Exercise: Is there any Difference in Common Symptoms and Quality of Life Among Female Patients with Rheumatic and Musculoskeletal Diseases? A Qualitative Study

**DOI:** 10.31138/mjr.161125.rad

**Published:** 2026-06-01

**Authors:** Dimitra K. Bonia, Anastasia Beneka, Asimenia Gioftsidou, Paraskevi Malliou

**Affiliations:** 1 Democritus University of Thrace, Department of Physical Education and Sports Science, Panepistimioupoli, Komotini, Greece;; 2 Metropolitan College, Department of Health Science, Athens, Greece

**Keywords:** rheumatic diseases, physical activity, exercise, quality of life, depression, interviews, women, qualitative research

## Abstract

**Aim:**

The purpose of this study was to explore female patients’ experiences regarding systematic exercise compared with low-intensity physical activity in relation to common symptoms and quality of life in RMDs.

**Methods:**

A qualitative study was conducted using semi-structured, face to face interviews. Fifteen women with RMDs were recruited; thirteen completed the interviews and were included in the analysis. Interviews lasted approximately 30 minutes and were audio-recorded. Data were analysed using Thematic Analysis following the six-phase approach of Braun and Clarke. Participants were categorised based on self-reported engagement in either systematic exercise or low-intensity physical activity (e.g. walking). NVIVO 15 software was used for data management and analysis.

**Results:**

Participants’ mean age was 58.3 ±13.38 years. Five a priori thematic areas were explored (pain, fatigue, depression, quality of life and exercise/physical activity), while an additional theme regarding barriers to participation emerged inductively. Most participants reported limited awareness of the distinction between physical activity and exercise. Women engaged in systematic exercise described more favourable experiences regarding symptoms management and quality of life, whereas those performing low-intensity physical activity primarily emphasised psychological benefits. Barriers to participation included physical limitations, fatigue, and motivational factors.

**Conclusion:**

From patients’ perspectives, systematic exercise and low-intensity physical activity are perceived differently in relation to symptom experiences and quality of life. These differences highlight the importance of individualised, patient-centred exercise counselling for women with RMDs.

## INTRODUCTION

Rheumatic and Musculoskeletal Diseases (RMDs) are chronic, predominantly autoimmune conditions characterised by persistent inflammation, leading to progressive damage and functional impairment. Although individual RMDs are relatively rare, collectively they affect a substantial proportion of the global population and represent a major public health burden. RMDs are associated with significant physical, psychological, and social consequences, resulting in impaired quality of life and mental health. Contemporary European and international evidence demonstrates that the impact of RMDs extends well beyond physical symptoms, encompassing depression, fatigue, reduced functionality, and limitations in daily and social participation.^1–2^

The pathophysiology of RMDs is primarily characterised by dysregulation of the immune system, leading to chronic autoimmune inflammation. This process involves activation of the innate immune response and classical immune pathways, resulting in the production of pro-inflammatory cytokines that act as key mediators in the initiation and maintenance of inflammation, ultimately contributing to tissue damage and disease progression.^4 –5^

Beyond immune dysregulation, increasing evidence suggests that the pathogenesis of RMDs is influenced by genetic susceptibility, which may be activated by environmental triggers such as infections, including viral agents.^6^ Furthermore, the adaptive immune response plays a central role, as reflected by the presence of disease-specific autoantibodies—such as anti-dsDNA, anti-citrullinated protein antibodies (ACPA), anti-PMScl, anti-Ku and anti-RNA polymerase III—in the serum of patients with RMDs.^7–8^ Among these, antinuclear antibodies (ANA) represent one of the most common immunological markers, indicating the presence of autoimmune inflammation.^9^

The symptoms of rheumatic and musculoskeletal diseases (RMDs) vary depending on the specific diagnosis and may include cardiovascular involvement, skin manifestations with various types of rashes, pulmonary fibrosis, Raynaud’s phenomenon, low-grade fever and joint deformities.^10–11^ Despite this clinical heterogeneity, several symptoms are consistently reported across patients with RMDs, with pain, severe fatigue and mild to moderate depressive symptoms, among the most common and burdensome manifestations.^12–14^

Furthermore, lifestyle-related factors such as obesity, physical inactivity, smoking, and alcohol consumption have been shown to negatively influence disease activity and symptom severity in RMDs, exacerbating inflammation, pain and psychological distress.^15^ An additional clinical feature observed mainly in rheumatoid arthritis is rheumatoid cachexia, characterised by inflammation-induced loss of muscle mass combined with increased adipose tissue, further aggravating functional impairment and the inflammatory burden of the disease.^16^

The management of RMDs primarily relies on pharmacological treatment, which aims to control disease activity and alleviate symptoms rather than provide a definitive cure. Standard pharmacological therapies include immunosuppressants, immunomodulatory agents, antimalarial drugs, and non-steroidal anti-inflammatory drugs, while more recent advances involve biologic and targeted synthetic therapies developed to reduce disease activity, comorbidities, and treatment-related adverse effects.^17–18^

Alongside pharmacological treatment, several non-pharmacological interventions have been implemented to manage symptoms and improve quality of life, including physiotherapy modalities (e.g. heat, cold, electrotherapy, and assistive devices), patient education, cognitive behavioural therapy (CBT), digital health and artificial intelligence–based applications, and structured exercise programs.^19^ Despite the documented benefits of these approaches, many patients with RMDs remain physically inactive. Evidence from a systematic review and meta-analysis of clinical trials indicates that exercise, particularly when combined with psychotherapeutic interventions, significantly reduces fatigue perception and overall symptom burden in patients with RMDs.^20^

In particular, higher levels of physical activity achieved through structured exercise have been shown to significantly improve the quality of life of patients with RMDs, especially with regard to physical pain, social participation, and functional capacity. Moreover, maintaining a healthy body weight in combination with regular exercise contributes to reduced joint damage and pain, while enhancing physical functioning in conditions such as ankylosing spondylitis (AS), osteoarthritis (OA), systemic lupus erythematosus (SLE), and systemic sclerosis (SSc).^21^

Additionally, exercise-based interventions have demonstrated beneficial effects on fatigue reduction and sleep quality improvement, particularly in patients with rheumatoid arthritis (RA). In contrast, low levels of physical activity, especially among older adults with RMDs, are associated with increased fatigue, persistent pain, sleep disturbances, and higher levels of depressive symptoms.^22^

More specifically, exercise exerts direct physiological and cellular effects by modulating immune system activity in patients with RMDs. Regular exercise is associated with a reduction in pro-inflammatory cytokines derived from adipose tissue and a concomitant increase in anti-inflammatory mediators, known as myokines, which are released by contracting skeletal muscle. Through the increased production of myokines, exercise promotes an anti-inflammatory immune profile, contributing to muscle regeneration and metabolic improvement. This mechanism is particularly relevant in counteracting rheumatic cachexia, a condition characterised by chronic inflammation-induced loss of skeletal muscle mass and increased adiposity.^23^

At this point, it is essential to clearly differentiate the term Physical Activity from Exercise. Physical Activity (PA) is defined as any bodily movement produced by skeletal muscles that results in energy expenditure and can be categorised into low-, moderate-, and high-intensity activity. PA includes a wide range of activities such as occupational tasks, household activities, recreational activities, walking, and exercise. In contrast, exercise is a subcategory of PA and differs in that it is planned, structured, repetitive, and performed systematically with the objective of maintaining or improving physical fitness.^24^

According to the EULAR recommendations, systematic exercise constitutes an effective non-pharmacological strategy for the management of fatigue in patients with rheumatic and musculoskeletal diseases.^25^ Furthermore, the World Health Organisation recommends that individuals with chronic conditions, including patients with RMDs, engage in low- to moderate-intensity physical activity for approximately 150–300 minutes per week, combined with resistance exercise two to three times per week.^26^

Walking, as a form of physical activity, is widely recommended for increasing overall PA levels, as it is strongly associated with improvements in physical function and quality of life, is easy to implement, and is cost-effective.^27^

Based on the evidence above, the aim of this study was to explore and analyse patients’ personal beliefs, perception, and expectation regarding systematic exercise and low-intensity PA, and whether participants perceived systematic exercise differently from low-intensity PA.

## METHODS

### Sample

The sample was consisted of women suffering from RMDs from Greece, whose condition had been certified by a rheumatologist, meeting the ACR classification criteria for each condition.^28^ The inclusion criteria were that the patients suffered from RMDs, were receiving medication for their condition, they were in remission and aged between 20 and 80 years old. The exclusion criteria were suffering from other conditions, e.g., cancer or multiple sclerosis, or receiving treatment for another condition not caused by the RMD they suffer from. To participate in the study, all the participants received written and verbal information about the study and provided written informed consent prior to participation. The study protocol was reviewed and approved by the Ethics and Deontology Committee of Democritus University of Thrace (Protocol No. APTH/EIDE/16171/158). The study conducted in accordance with the principles of the Declaration of Helsinki (December 2024 revision). The sample was approached via an e-mail of the official associations of Rheumatism Patients EL.E.A.NA and the Association of Rheumatism Patients of Western Attica “IISO”. Sampling took place from 1^st^ of July, to 31st of July, and involved face-to-face interviews with female patients who were randomly selected by the official associations. Given the qualitative and exploratory nature of the study, the sample size and age range were not intended to support generalisation or age-based comparisons. The aim was to capture in-depth perceptions across a heterogeneous group of women with RMDs rather than to examine age- or diagnosis-specific differences.

### Procedure

Fifteen female patients were initially interviewed. Two interviews were excluded from the final analysis due to the early termination and incomplete data, resulting in a final sample of thirteen participants, to collect qualitative data based on the biopsychosocial model,^29^ with questions addressing issues related to their personal opinions, beliefs, and their expectations regarding systematic exercise or low-intensity physical activity (walking), in relation to pain, fatigue, depression, and quality of life, as well as factors that influence their participation. The interview lasted approximately 25 to 30 minutes for each participant and was conducted in July 2025. The questions were selected from evidence-based qualitative studies that had been conducted on the respective topics in RMDs, such as RA.^30–31^ To enhance the reliability and clarity of the interview questions, a pilot test was conducted on two patients with RMDs, assessing comprehension, variety of responses, and the logical sequence of questions.

### Interview

Regarding the qualitative analysis of the study, demographic data were collected from the participants, followed by semi-structured interviews aimed at gaining a deeper understanding of the views of patients with RMDs and their relationship with systematic exercise or low-intensity physical activity. The analysis of the interviews was based on a phenomenological approach, which focuses on the empirical understanding of how respondents experience a particular phenomenon,^32^ in this study, systematic exercise or low-intensity physical activity. All interviews were conducted face-to-face by a single researcher who had received formal training in qualitative research methods prior to data collection. The interviewer followed a structured semi-structured interview guide, which was developed based on previous qualitative studies in patients with RMDs and was pilot tested to the main study. The research process was conducted under academic supervision by senior researchers in qualitative research and Thematic Analysis. The interviewer had no prior relationship with the participants before the interviews were conducted. To minimise interviewer bias, the same interview was used for all participants, and neutral-ended questions were employed throughout the interviews. This Qualitative study was reported in accordance with the Consolidated Criteria for Reported Qualitative Research (COREQ) guidelines (**Appendix 1**).

For the thematic analysis, the methodological approach of Braun & Clarke was used, which followed the six stages of the approach: (1) Familiarisation with the data, (2) Initial coding, (3) Searching for themes, (4) Reviewing themes, (5) Defining themes, and finally (6) Writing the analysis and the results. However, the themes that emerged were created from the initial variables of pain, fatigue, depression, and quality of life. Coding was inductive and based on the participants’ responses. To ensure validity, representative verbatim quotations were used to support the identified themes.^33^ Through thematic analysis and the initial variables, the data were organised into four main domains concerning pain, fatigue, depression, and quality of life, while a fifth theme emerged concerning factors influencing participation in activities and experience. Furthermore, theoretical triangulation of sources was applied to highlight similarities and differences between participants, with the creation of two groups, the group of the Systematic Exercise and the Low-intensity Physical Activity, from the same sample.^34^ The categorisation of participants into those engaging in systematic exercise and those performing low-intensity physical activity was used solely as a descriptive and organisational framework to support thematic interpretation. This categorisation does not constitute a formal subgroup analysis and was not intended to allow comparative or inferential conclusions. For the analysis of the qualitative data NVIVO 15 was used.

## RESULTS

A total of 13 women suffering from various RMDs participated in the study, with a mean age of 58.38 (SD=13.38). Of the participants 46.5% (n=6) had been diagnosed with RA, 23.8% (n=3) with SLE, and 30.77% (n=4) suffered from another RMDs (Rupus, SSC, AS). Regarding place of residence, 53.85% (n=7) responded that live in a large city, 30.77% (n=4) in a small town, while 15.38% (n=2) responded that they live in a village. Of the total sample of 13 responders, 61.54% (n=8) responded that they were not working, while the remaining 38.46% (n=5) responded that they are employed. Finally, 69.23% (n=9) responded that they participated in low-intensity physical activity (daily household chores & walking), 30.77% (n=4) stated they participated in weekly systematic exercise under supervision of a professional trainer, while 90% (n=12) responded that they were not aware of the conceptual distinction between physical activity and exercise prior to the interview. For descriptive purposes, participants were categorised according to their reported activity patterns, by dividing the participants into two groups according to their responses, the low-intensity physical activity (daily walking), and the systematic exercise group (**Table 1**). Participants who reported engaging in systematic exercise described their experiences in relation to pain, fatigue, emotional state, and quality of life. Several participants reported perceived changes in pain intensity during daily life, while during periods of symptom exacerbation they described choosing lighter forms of activity, such as walking or stretching, in order to remain active. In relation to fatigue, participants described systematic exercise as being associated with temporary changes in energy levels. Some participants reported feelings of rejuvenation following exercise sessions, while others emphasised the importance of regulating exercise intensity in order to avoid excessive exhaustion. Regular participation in exercise was described as requiring planning and consistency.

**Table 1.** Demographic characteristics of the sample.

**Demographic Data**	**Responses**	**% (n=13)**
Disease	RA	46,15% (n=6)
	SLE	23,08% (n=3)
	Other	30,77% (n=4)

**Residence**	City	53,85% (n=7)
	Town	30,77% (4=)
	Village	15,38% (n=2)

**Occupation Status**	Employed	38,46% (n=5)
	Unemployed/Retired	61,54% (=8)

**Activity Preference**	Low-intensity Physical	69,23% (n=9)
	Activity	
	Systematic Exercise	30,77% (n=4)

**Knowledge between Exercise or Physical Activity difference**	Yes	10% (n=1)
	No	90% (n=12)

With regard to emotional well-being, participants engaging in systematic exercise frequently described exercise as a means of emotional expression and stress relief. Several participants associated exercise with enhanced self-image and feelings of self-efficacy. In terms of quality of life, participants described associations between systematic exercise and perceptions of increased independence, social participation, and daily functioning. Exercise programs were described as individualised and typically included aerobic, resistance, and flexibility components, with a duration of approximately 60 minutes per session, performed two to four times per week. Despite ongoing participation, participants also described barriers to systematic exercise, including joint stiffness, financial cost, and limited available time (**Table 2**).

**Table 2.** Comparison of thematic analysis.

**Thematic Categories**	**Systematic Exercise Group**	**Reference of Systematic Exercise Group**	**Low-intensity Physical Activity Group**	**Reference of Low-intensity Physical Activity Group**
**Effect of Exercise or PA on Pain**	Reduced pain, mainly on joints, with positive effects	“When I move, I forget the pain” (Patient 8)	Increased pain as a deterrent factor	“When I feel pain I stop everything” (Patient 1)
**Effect of Exercise or PA on fatigue**	Reduced fatigue, and increased effect on activation	“Exercise fills me with energy. I start the day more powerful (Patient 12)	Increased severe fatigue as a deterrent factor	“I feel terrible fatigue, I want to lie down immediately.” (Patient 13)
**Effect of Exercise or PA on depression/Mental Health**	Mental empowerment	“My mind is peaceful during exercise” (Patient 6)	Increased need for social isolation, mild depression	“Psychologically speaking I feel down because I don’t have QoL.” (Patient 13)
**Effect of Exercise or PA on QoL**	Feeling of autonomy and independence	“I don’t want to be depended; exercise keeps me functional” (Patient 10)	Low QoL, beliefs that exercise won’t help	“I can’t perform at the same level as before, and that’s bother me” (Patient 4)
**Influencing Factors/Obstacles**	Mainly the symptoms of joint stiffness, financial burden, limited time	“Some days are difficult but I keep trying (Patient 6)	Fear of exacerbation, lack of motivation and guidance.	“None have ever mentioned to me what to do or if I should do “ (Patient 5)
Ideal exercising program	Daily base morning exercise, with tailored exercises	“I start with some stretching, and I continue with fast walking till I start sweating” (Patient 12)	Occasional desire for some gentle activities such as walking	“Waking might be helpful, but not everyday” (Patient 3)

This comparison is between the group of systematic exercise and the group of low-intensity physical activity regarding the symptoms of pain, fatigue, depression, levels of QoL, the obstacles of absence of exercise, and the activity preference.

Participants who reported engaging primarily in low-intensity physical activity, such as walking, described varied experiences related to pain, fatigue, emotional state, and quality of life. Some participants reported temporary perceived changes in pain following activity, while others described a rapid return or worsening of symptoms after activity. Fatigue was frequently described as a dominant factor influencing activity levels, with several participants reporting exhaustion following physical activity and others describing the need to manage fatigue through rest or activity avoidance.

In relation to quality of life, participants engaging in low-intensity physical activity described the impact of symptoms on daily functioning and psychological well-being. Although the importance of remaining physically active was acknowledged, many participants reported difficulties incorporating systematic exercise into their daily routines. Low-intensity physical activity was described as typically consisting of walking lasting approximately 19 to 25 minutes, performed one to two times per week. Reported barriers included pain, fatigue, fear of symptom exacerbation, lack of motivation, and limited access to facilities with qualified exercise professionals.

**Figure 1.**
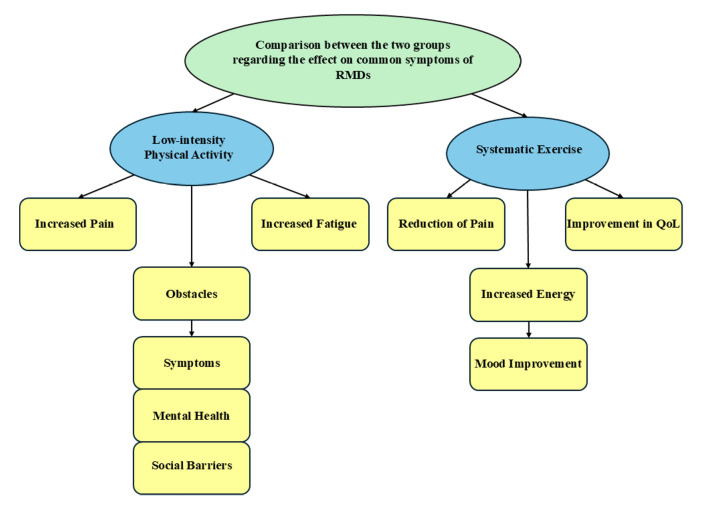
Thematic map illustrating the effect of systematic exercise and low-intensity physical activity on each group.

Across the interviews, narrative contrasts emerged between participants engaging in systematic exercise and those engaging primarily in low-intensity physical activity. Participants engaging in systematic exercise more frequently described structured guidance, planning, and emotional benefits, whereas participants engaging in low-intensity physical activity more often emphasised symptom burden, fatigue, and lack of professional support. These contrasts reflect participants’ reported experiences and perceptions and are presented descriptively rather than as formal comparative findings (**Figure 1**).

## DISCUSSION

This qualitative study explored how women with rheumatic and musculoskeletal diseases perceive systematic exercise and low-intensity physical activity in relation to pain, fatigue, depressive symptoms, and quality of life. The findings should be interpreted as perception-based and experiential, rather than as evidence of objective clinical effects. Participants who reported engaging in systematic exercise consistently described perceived symptom relief, improved mood, and enhanced functionality, whereas those engaging primarily in low-intensity physical activity emphasised persistent symptoms and greater perceived barriers to participation. These narrative contrasts reflect differences in personal experiences and attitudes toward movement, rather than measurable differences in clinical outcomes.

On the other hand, low-intensity physical activity group appeared to have more persistent pain and fatigue. This leads to reduced participation, and symptoms were managed by avoiding movement and increased the need for immediate rest, which may negatively affect perceived quality of life.

The biggest difference between the two groups was how they dealt with and behaved towards the symptoms and the condition itself, and how they felt through increased mobility. The group of systematic exercise did not consider pain as a barrier, as much as the low-intensity physical activity group, their perceptions and responses to pain. In the systematic exercise group, despite the barrier of economic burden, limited time or the symptoms themselves, it was found that being more active reduces the symptoms more effectively than resting. In contrast, low-intensity physical activity group was driven to hypomobility.

The patients between the two groups had a different point of view in the perception of pain, and their symptoms in general. The systematic exercise group had a more positive attitude despite their pain, which led them to mobility, better mental state, and independence.

According to the EULAR recommendations and the World Health Organisation guidelines, systematic exercise, increased levels of physical activity, and avoidance of sedentary behaviour are strongly recommended, particularly for patients with RMDs.^35–36^ Exercise is considered a multifactorial and beneficial intervention for overall health, with well-documented positive effects on pain, fatigue, and physical functioning, while sedentary behaviour has been consistently associated with poorer quality of life.

Fatigue represents one of the most persistent and burdensome symptoms reported by patients with RMDs. Evidence from a systematic review indicates that patients who engage in higher levels of physical activity experience significantly lower levels of fatigue compared to those with a sedentary lifestyle, although distinctions between structured exercise and low-intensity physical activity were not always clearly defined.^37^ Improvements in pain and general health through exercise as a therapeutic intervention have been consistently reported in patients with rheumatoid arthritis (RA), with significant positive effects on pain, fatigue, depressive symptoms, and overall quality of life.^38^

In addition, low-intensity physical activities such as walking, yoga, and Tai Chi have been shown to significantly improve mental health, sleep quality, cognitive performance, physical functioning, and quality of life in patients with fibromyalgia syndrome (FMS).^26^ Furthermore, patients with RA who regularly engage in low-intensity daily activities—including household tasks, walking, and gardening—demonstrate lower levels of disease activity, pain, fatigue, and anxiety compared to more sedentary individuals.^27^

Barriers to participation in exercise programs among patients with rheumatic and musculoskeletal diseases are primarily related to disease-related symptoms, particularly in conditions such as systemic sclerosis (SSc), where pain, fatigue, and functional limitations significantly restrict engagement in physical activity.^39^ Nevertheless, the need for structured guidance and supervision from healthcare professionals is widely acknowledged and should be considered an integral component of rehabilitation programs, especially for patients with rheumatoid arthritis (RA).^40^ Furthermore, growing evidence emphasises the importance of personalised exercise interventions, highlighting that healthcare professionals implementing systematic exercise should actively consider patients’ individual preferences, capabilities, and perceived barriers in order to optimise adherence and long-term effectiveness.^41^ Several limitations should be considered when interpreting the findings of this study. The small sample size and wide age range limit the transferability of the results and do not allow age-specific interpretations. In addition, the inclusion of women with different rheumatic and musculoskeletal diseases introduces clinical heterogeneity in terms of symptom burden, disease duration, and progression, which were not systematically analysed. Participants were recruited through patient associations using voluntary response, which may have favoured individuals who were more motivated, socially engaged, or interested in physical activity, introducing potential selection bias. Finally, the categorisation into activity-based groups was descriptive and exploratory, and should not be interpreted as a comparative subgroup analysis.

## Data Availability

Due to the qualitative nature of this study and the sensitive personal data shared during in-depth interviews, the datasets generated and/or analysed are not publicly available in order to protect participant confidentiality. Anonymised data excerpts supporting the findings of this study are available from the corresponding author upon reasonable request and in accordance with ethical approval.

## DECLARATIONS

This study was conducted in accordance of Declaration of Helsinki, and ethical approval was obtained from the appropriate Institutional Review Bord and Ethics Committee of Democritus University of Thrace. All participants provided written informed consent prior to their inclusion to the study.

## CONFERENCE PRESENTATION

Preliminary findings from this study were presented as a conference abstract at the 5^th^ Panhellenic Conference of Functional Management and Physical Active Individuals (LEI.DI.A.A.,2025), Greece. The abstract has not been published as a full paper and no DOI is available.

## EDITING SUPPORT AND AI USE

No professional editing agency was used for the presentation of this manuscript.

Artificial intelligence-based tools were used for language refinement and clarity. All content was reviewed, edited, and approved by the authors, who take full responsibility for the accuracy, originality, and integrity of the manuscript.

## APPENDIX

https://mjrheum.org/assets/files/supplementary/MJR%202025-0414_APPENDIX.pdf
